# Targeting the CD47–SIRPα phagocytic checkpoint in cancer: Biology, translational opportunities, and next‐generation therapeutic strategies

**DOI:** 10.1002/smo2.70079

**Published:** 2026-07-15

**Authors:** Ruimei Zhou, Lingjie Jing, Jianping Zhang, Cheng Guo, Quanjun Yang

**Affiliations:** ^1^ Department of Pharmacy Shanghai Sixth People's Hospital Affiliated Shanghai Jiao Tong University School of Medicine Shanghai China; ^2^ College of Clinical Pharmacy Shanghai Jiao Tong University School of Medicine Shanghai China

**Keywords:** CD47, immunotherapy, innate immunity, phagocytic checkpoint

## Abstract

Immune checkpoint inhibitors have transformed cancer therapy with the programmed cell death protein 1–programmed death‐ligand 1 axis, demonstrating substantial efficacy by targeting adaptive immunity across multiple malignancies. However, the limited clinical responses observed in a considerable proportion of patients highlights the need for more effective engagement of innate immune mechanisms. In this context, the cluster of differentiation 47–signal regulatory protein α (CD47–SIRPα) axis has emerged as a next‐generation immune checkpoint that regulates phagocytosis. CD47 is a ubiquitously expressed transmembrane glycoprotein containing an N‐terminal extracellular immunoglobulin variable‐like domain and is frequently overexpressed in both solid tumors and hematological malignancies. By binding to SIRPα on macrophages, CD47 transmits a canonical “don't eat me” signal that suppresses phagocytosis and enables tumor immune evasion. Beyond this canonical role, CD47 interacts with ligands such as thrombospondin‐1 and integrins to regulate tumor cell migration, metabolic adaptation, and immune balance within the tumor microenvironment. Importantly, CD47 links innate immune clearance with antigen presentation and downstream adaptive immune activation, positioning it as an actionable node for next‐generation therapeutic designs. Although the first‐generation CD47 blockade has revealed challenges related to hematologic toxicity, antigen sink effects, and limited monotherapy durability, these challenges have also accelerated the development of more selective, controllable, and context‐responsive therapeutic strategies. In this Review, we elucidate the structural features, molecular mechanisms, pathological functions, therapeutic strategies, translational challenges, and emerging solutions of CD47, with the aim of providing a theoretical basis for overcoming current therapeutic limitations and advancing more precise cancer immunomodulatory strategies.

## Graphical abstract

The CD47–SIRPα axis has emerged as a critical innate immune checkpoint that suppresses macrophage‐mediated phagocytosis through a canonical “don't eat me” signal and enables tumor immune evasion. We comprehensively summarize the structural and biological features of CD47 and the molecular mechanisms underlying CD47‐mediated regulation of phagocytosis. By discussing the pathological roles of CD47 in tumor progression and immune evasion, we provide an integrated overview of current therapeutic strategies targeting CD47, highlight their evolution from first‐generation blockade to second‐generation and third‐generation intelligent therapeutic designs, and critically evaluate their clinical progress, opportunities, and limitations.

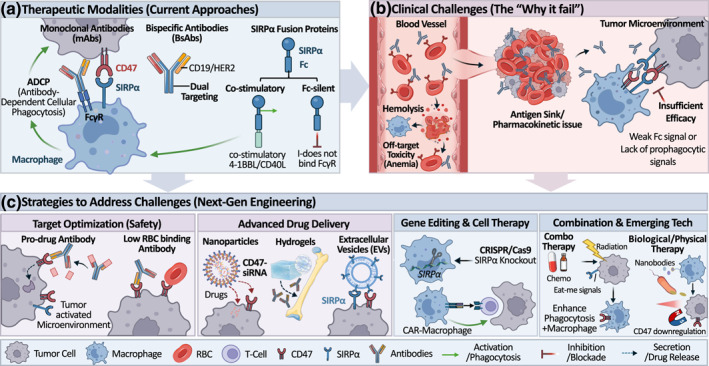

## 1 INTRODUCTION

Immune checkpoint blockade restores antitumor immunity by reversing tumor‐induced immunosuppression. Conventional checkpoints, including programmed cell death protein 1–programmed death‐ligand 1 (PD‐1–PD‐L1) and cytotoxic T lymphocyte‐associated protein 4 axes, primarily act on T cell‐mediated adaptive immunity and significantly improve survival across multiple cancer types. Nevertheless, overall response rates remain limited and resistance is common, indicating that T cell‐focused activation alone is insufficient to overcome all immune evasion mechanisms1.^[1]^ In recent years, increasing attention has been directed toward the concept of innate immune checkpoints, highlighting how tumors evade immune clearance by suppressing phagocytosis and antigen presentation by myeloid cells, including macrophages, dendritic cells (DCs), and neutrophils.^[2]^ Among these pathways, the CD47–SIRPα axis is regarded as a prototypical phagocytic checkpoint, whereby tumor cells overexpress CD47 to deliver a “don't eat me” signal, thereby inhibiting antibody‐dependent cellular phagocytosis (ADCP) and basal innate engulfment.^[3]^

CD47 is a ubiquitously expressed transmembrane protein that binds to the inhibitory receptor SIRPα in myeloid cells. This interaction induces immunoreceptor tyrosine‐based inhibitory motif (ITIM) phosphorylation and recruitment of Src homology region 2 domain‐containing phosphatase‐1/2 (SHP‐1/2) phosphatases, ultimately suppressing cytoskeletal rearrangements required for phagocytosis.^[4]^ Beyond directly relieving innate immune inhibition, blockade of the CD47–SIRPα axis may enhance tumor antigen uptake and cross‐presentation, thereby facilitating T cell priming and amplifying adaptive immune responses through an innate‐to‐adaptive immune cascade.^[5]^ This property makes CD47 an attractive strategy for complementing the T cell‐centered checkpoint blockade, particularly in immunologically “cold” or myeloid‐dominant tumor microenvironments.[^3^, ^5^, ^6^]

Despite its strong therapeutic rationale, CD47 targeting presents an inherent therapeutic window challenge. CD47 is highly expressed on erythrocytes, platelets, and hematopoietic cells and plays a fundamental role in self‐recognition and homeostasis.^[7]^ It is frequently upregulated in both solid and hematological malignancies, and is associated with invasion, metastasis, stemness, and poor prognosis.^[8]^ This dual expression pattern gives rise to two major obstacles: hematologic toxicities, including anemia and thrombocytopenia driven by fragment crystallizable (Fc)‐mediated effects, and antigen sink–mediated drug sequestration by peripheral blood cells, which limits tumor exposure and necessitates dose escalation.[^9^, ^10^] Clinical setbacks, including the Food and Drug Administration hold on magrolimab programs in hematologic malignancies, further underscoring the need to balance efficacy, safety, and tumor selectivity.^[11]^ Accordingly, current strategies focus on affinity tuning, Fc engineering, bispecific formats, and targeted delivery to improve tumor selectivity, while minimizing systemic toxicity.^[4]^ Thus, the CD47 field has shifted from proof‐of‐mechanism to the challenge of engineering clinically translatable and reproducible therapeutic solutions.^[10]^

Against this background, CD47 is increasingly being viewed as a platform for intelligent therapeutic design and not merely as a target for blockade. In this review, we systematically summarize the structural features and post‐translational modifications (PTMs) of CD47, its ligand interactions and physiological functions, and its pathological roles in tumor immune evasion and disease progression. We further integrated current CD47‐targeting strategies, key challenges in clinical development, and emerging solutions from the perspectives of drug engineering and smart therapeutic systems. By doing so, we aimed to provide an integrated framework spanning molecular mechanisms and clinical translation, while informing rational drug design, combination strategies, and biomarker development targeting the CD47 axis.

## 2 BIOLOGICAL PROPERTIES AND STRUCTURE OF CD47

### 2.1 Molecular architecture and functional domains of CD47

**FIGURE 1:**
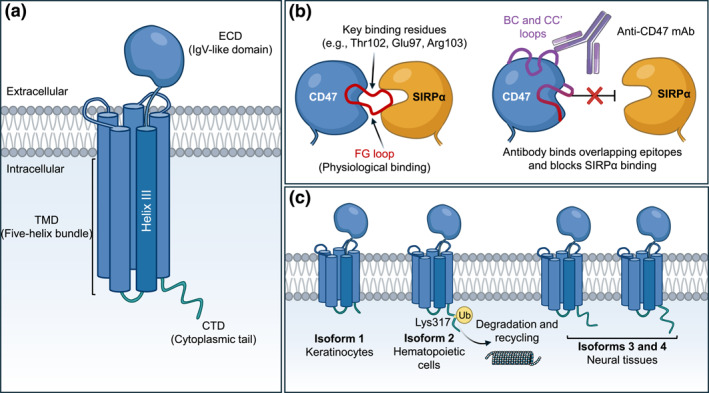
Structural topology and isoform diversity of CD47. (a) Overall topology of CD47. CD47 comprises an extracellular IgV‐like domain, a five‐transmembrane helical bundle, and a C‐terminal cytoplasmic tail. (b) CD47–SIRPα recognition and therapeutic blockade. The ECD of CD47 engages SIRPα mainly through the FG loop, with key residues spanning Glu97 to Arg103. Therapeutic anti‐CD47 monoclonal antibodies recognize epitopes within the BC and CC’ loops, thereby sterically blocking the CD47–SIRPα interaction. (c) Isoform diversity of CD47. Alternative splicing generates tissue‐biased CD47 isoforms with distinct cytoplasmic tails, which may contribute to isoform‐specific receptor homeostasis and regulation. ECD, extracellular domain.

CD47, also known as integrin‐associated protein, is a ubiquitously expressed cell‐surface glycoprotein with an apparent molecular weight of approximately 47–52 kDa. Structurally, CD47 is a unique five‐transmembrane protein composed of an N‐terminal extracellular immunoglobulin variable‐like (IgV‐like) domain, a multi‐pass transmembrane domain (TMD), and a short cytoplasmic tail that varies among isoforms owing to alternative splicing^[12]^ (Figure 1a).

*IgV‐like extracellular domain*: The IgV domain forms the core interface, through which CD47 mediates self‐recognition. Structural studies have shown that CD47–SIRPα binding is governed by multiple surface loops, among which the FG loop contributes the most strongly to binding energy and interface complementarity. In the CD47–SIRPα complex (PDB ID: 2JJT), FG‐loop residues such as Thr102 are inserted into the SIRPα D1 binding groove, while clustered hot spot residues including Glu97, Thr99, Glu100, and Arg103 further stabilize the interaction through electrostatic complementarity, providing defined targets for epitope engineering and inhibitor design[^13^, ^14^] (Figure 1b). Structural analyses of CD47–magrolimab and CD47–B6H12.2 complexes further indicated antibody binding to residues such as Tyr37 and Asp46 within the BC and CC’ loops, which overlap with the SIRPα‐binding epitope and explain their blocking activity, while highlighting the importance of epitope selection and affinity tuning for the therapeutic window and antigen sink effects[^15^, ^16^] (Figure 1b).

*Five‐transmembrane helical bundle*: The transmembrane region of CD47 comprises five hydrophobic helices that assemble into a tightly packed helical bundle, a feature unusual within the immunoglobulin superfamily. Structural data indicate that these helices create a hydrophobic core, with helix III occupying a central position and engaging in multiple stabilizing interactions with neighboring helices. Notably, conservation mapping revealed a continuously conserved surface extending from the SIRPα‐binding interface on the extracellular domain (ECD) into the transmembrane region, suggesting structural coupling between extracellular ligand recognition and transmembrane conformation or stability.^[12]^ Functionally, the TMD not only anchors and stabilizes the membrane‐proximal orientation of the ECD but also serves as an organizational hub for multiprotein complexes, facilitating the coupling of extracellular ligand engagement with membrane‐associated rearrangements or intracellular signaling events.^[17]^

*Cytoplasmic tail domain and alternative splicing*: The cytoplasmic tail of CD47 was relatively short. However, alternative splicing determines its length and sequence diversity, generating tissue‐specific expression patterns and conferring distinct intracellular regulatory potential. Isoform repertoires vary across species, and are primarily produced by exon skipping.^[18]^ In humans, the CD47 cytoplasmic tail domain (CTD) is encoded by 13 exons and gives rise to four major splice isoforms (isoforms 1–4) with distinct tissue distributions, indicating functional specialization. Isoform 1 is enriched in keratinocytes and certain tumors,^[19]^ and isoform 2 is the most widely expressed and is prevalent in hematopoietic, endothelial, and epithelial cells, with reported roles in astrocyte extracellular matrix–cytoskeleton signaling.^[20]^ In contrast, isoforms 3 and 4 are relatively enriched in neural tissues and correlate with learning‐ and memory‐related parameters, suggesting their involvement in memory consolidation^[21]^ (Figure 1c). Different CTDs differentially influence interactions with intracellular adaptor proteins, thereby affecting receptor trafficking, endocytosis, homeostasis, and signaling bias. Longer tails contain key ubiquitination sites, such as lysine 317, which participate in receptor degradation and recycling and modulate immunoregulatory outcomes.^[22]^ This organization implies that CD47 extracellular blocking epitopes are relatively conserved, whereas the intracellular regulatory modules are highly heterogeneous.

### 2.2 PTMs of CD47

**FIGURE 2:**
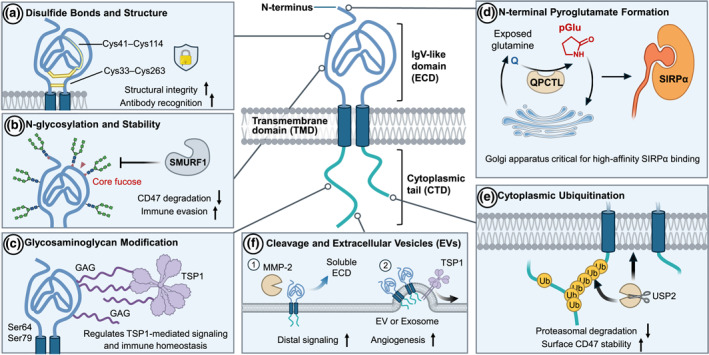
Post‐translational modifications governing CD47 structural integrity and functional regulation. (a) Disulfide bond stability. Disulfide bonds preserve the integrity of the extracellular domain and support antibody recognition. (b) N‐glycosylation and receptor stability. Tumor‐associated glycosylation, including core fucosylation, regulates CD47 degradation and immune‐evasive function. (c) GAG modification. GAG chain attachment at defined extracellular residues converts CD47 into a proteoglycan‐like state, thereby modulating TSP1‐dependent signaling and immune regulation. (d) Pyroglutamate formation. QPCTL catalyzes pGlu formation at the CD47 N‐terminus, supporting high‐affinity SIRPα binding. (e) Ubiquitination regulation. USP2‐mediated deubiquitination regulates CD47 proteasomal degradation and surface stability. (f) Proteolytic shedding and vesicle transfer. CD47 can be released from the cell surface as a soluble extracellular fragment or transported with TSP1 in extracellular vesicles, thereby extending CD47‐associated signaling beyond the plasma membrane. GAG, glycosaminoglycan.

The biological activity of CD47 is regulated by multiple PTMs. These modifications affect the receptor folding, membrane stability, ligand recognition, and antibody binding. Disulfide bonds maintain the conformation of the extracellular IgV‐like domain and support its coupling to the transmembrane region. Disruption of these bonds impairs antibody recognition, cell adhesion, and downstream signaling, highlighting their importance in preserving the functional architecture of CD47[^23^, ^24^] (Figure 2a). Glycosylation further shapes the structural stability and immune recognition properties of CD47. The N‐terminal IgV‐like domain contains multiple N‐glycosylation sites, and aberrant tumor‐associated glycosylation may influence CD47 trafficking, degradation, and ligand binding.^[25]^ Recent studies have shown that core fucosylation suppresses SMAD‐specific E3 ubiquitin protein ligase 1‐mediated degradation, thereby increasing CD47 expression.^[26]^ In bladder cancer, site‐specific glycosylation enhances CD47 binding to SIRPα and may promote tumor immune evasion^[27]^ (Figure 2b). Together, these findings indicate that CD47 function is determined not only by its expression level but also by the conformation and biochemical state of its ECD.

In addition to maintaining structural stability, specific PTMs can directly regulate CD47‐mediated immune signaling. Glycosaminoglycan modification converts CD47 into a proteoglycan‐like form and modulates thrombospondin‐1 (TSP1)‐dependent T cell receptor (TCR) signaling. In parallel, T cell activation can induce the synthesis and release of CD47 isoforms, which may regulate TSP1–CD47 signaling[^28^, ^29^] (Figure 2c). However, these mechanisms have been mainly defined in vitro. Whether they are further shaped by microenvironmental context or ligand availability in complex tissues remains to be clarified. N‐terminal pyroglutamate (pGlu) formation provides a more direct mechanism for controlling the CD47–SIRPα interaction interface. After signal peptide cleavage, glutaminyl‐peptide cyclotransferase‐like protein (QPCTL) catalyzes the conversion of the exposed N‐terminal glutamine into pGlu. Inhibition of QPCTL reduced the binding of CD47 to SIRPα‐Fc fusion proteins without markedly decreasing total cell‐surface CD47 expression[^30^, ^31^] (Figure 2d). These findings suggest that interfering with receptor maturation, rather than directly blocking extracellular epitopes, can weaken functional CD47–SIRPα recognition. Although this strategy remains exploratory, it may help to broaden the hematologic safety window of CD47‐targeted therapy by selectively attenuating checkpoint engagement.

Regulation of CD47 homeostasis and extracellular processing further expands the functional scope of this immune checkpoint. Deubiquitinase ubiquitin‐specific peptidase 2 removes lysine 48 (K48)‐linked ubiquitin chains from CD47, thereby limiting proteasomal degradation and stabilizing cell‐surface expression. USP2 inhibition decreased CD47 levels, enhanced macrophage phagocytosis, and promoted the polarization of tumor‐associated macrophages (TAMs) toward an M1‐like pro‐inflammatory phenotype^[32]^ (Figure 2e). These results suggest that intracellular control of CD47 stability may complement extracellular CD47–SIRPα blockade. In addition, CD47 function is not restricted to an intact plasma membrane. Its ECD can be proteolytically cleaved and released by enzymes such as matrix metalloproteinase‐2, and this process is influenced by metabolic conditions.^[33]^ Extracellular vesicles (EVs) can also transfer CD47 and TSP1 to recipient cells, thereby modulating immune responses and endothelial cell behavior^[34]^ (Figure 2f). Clinically, EV‐associated CD47 has been linked to a poor prognosis and immunosuppressive circuits. This suggests that EV‐associated CD47 should be considered when evaluating CD47‐targeted interventions, particularly with respect to antigen sink effects, drug distribution, and immunotherapeutic outcomes.^[35]^ Collectively, these processes reveal the multilayered regulation of CD47 within the immune microenvironment and provide a conceptual basis for future translational strategies targeting CD47‐dependent immune evasion.

## 3 LIGANDS AND PHYSIOLOGICAL FUNCTIONS OF CD47

### 3.1 Major ligands and interaction networks

CD47 engages in a diverse ligand repertoire, which can be broadly classified into immunoinhibitory receptors, extracellular matrix ligands, and adhesion molecule networks. Through these multilayered interactions, CD47 integrates functions in setting phagocytic thresholds, coordinating tissue repair, regulating metabolism, and remodeling cell migration, thereby serving as a central hub for immune and tissue homeostasis.

**FIGURE 3:**
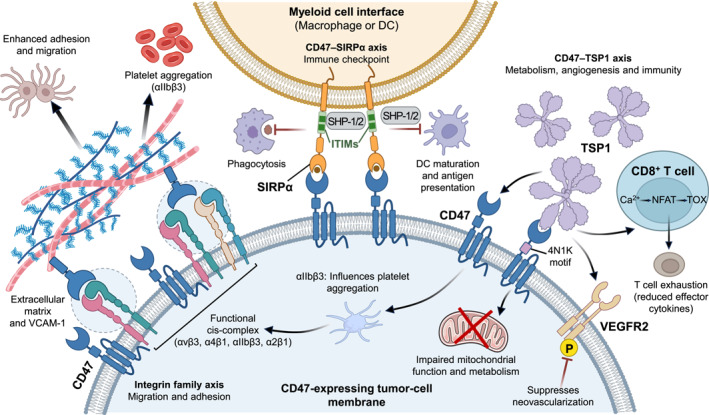
Major ligand networks and physiological functions of CD47. CD47 integrates SIRPα‐, TSP1‐, and integrin‐dependent signaling to coordinate phagocytic inhibition, antigen presentation, angiogenic control, metabolic regulation, cell adhesion, and T cell functional states.

*SIRPα*: SIRPα is a transmembrane inhibitory receptor that is highly expressed in myeloid cells and consists of three extracellular Ig‐like domains, a transmembrane region, and a cytoplasmic tail that contains ITIM motifs. Its N‐terminal V‐like domain mediates specific binding to CD47, and ligand engagement recruits SHP‐1/2 phosphatases to suppress phagocytosis, forming a core component of self‐recognition mechanisms.[^9^, ^17^] Beyond phagocytosis, CD47–SIRPα signaling modulates monocyte‐derived DC maturation and inflammatory responses, and reduces their capacity to prime T cells, implicating this axis in antigen presentation and immune initiation.^[36]^ Further evidence indicates that CD47–SIRPα signaling interferes with the cytosolic sensing of tumor‐derived DNA in DCs and downstream immune activation, contributing to myeloid suppression and defective antigen presentation in tumors[^5^, ^37^] (Figure 3).

*TSP1*: TSP1 is a secreted extracellular matrix glycoprotein that binds to CD47 via its C‐terminal 4N1K motif and regulates multiple physiological processes. CD47‐dependent TSP1 signaling impairs mitochondrial function and metabolic homeostasis, and clinical studies have associated aberrant TSP1 expression with obesity, fatty liver disease, and diabetes.^[38]^ In angiogenesis, TSP1–CD47 interactions inhibit vascular endothelial growth factor receptor 2 (VEGFR2) phosphorylation and suppress neovascularization, a mechanism that has been extensively validated in wound healing and vascular homeostasis.^[39]^ Within the TME, TSP1–CD47 signaling activates the Ca^2+^‐nuclear factor of activated T cells (NFAT)‐thymocyte selection‐associated high mobility group box protein (TOX) pathway in MC38 colorectal cancer and B16 melanoma tumor models, thereby driving CD8^+^ T cell exhaustion and reducing effector cytokine production. However, its specific clinical translatability remains to be further explored^[40]^ (Figure 3). In addition, when coupled with the CD47–SIRPα axis, TSP1–CD47 signaling can simultaneously regulate T cell functional states and macrophage phagocytic thresholds, providing a conceptual basis for combinatorial targeting strategies, although this direction remains at an early exploratory stage.^[41]^

*Integrin family*: Integrins are heterodimeric transmembrane receptors composed of α‐ and β‐subunits with specific subunit combinations that mediate distinct biological functions. Substantial evidence indicates that CD47 forms functional membrane complexes with multiple integrins, including αvβ3, α4β1, αIIbβ3, and α2β1, and modulates adhesion‐ and migration‐related phenotypes.^[42]^ Furthermore, CD47 regulates α4β1‐mediated adhesion and its interactions with ligands such as vascular cell adhesion molecule 1. This mechanism has been demonstrated in in vitro adhesion assays using isolated platelets and erythrocytes under α4β1 activation conditions, highlighting the functional role of CD47 in adhesion dynamics. However, this effect is highly dependent on experimental conditions and ligand presentation.^[43]^ In platelets, CD47–TSP1 interactions modulate αIIbβ3 activity and aggregation, and both in vitro experiments and animal studies have indicated that CD47 plays an important role in maintaining thrombotic homeostasis^[44]^ (Figure 3). Recent studies have shown that stable interactions between CD47 and αvβ3 in tumor cells promote immune evasion.^[45]^ However, this pathway remains at an early exploratory stage, as current evidence is derived mainly from murine xenograft models and in vitro CD8^+^ T cell experiments, leaving its generalizability and clinical translatability within complex tumor microenvironments unclear.

### 3.2 Intracellular signaling and immune balance

CD47 exerts pleiotropic intracellular effects through cooperation with molecules involved in metabolism, cytoskeletal dynamics, and membrane microdomains, thereby modulating cell survival, migration, and immune activation thresholds.^[20]^

**FIGURE 4:**
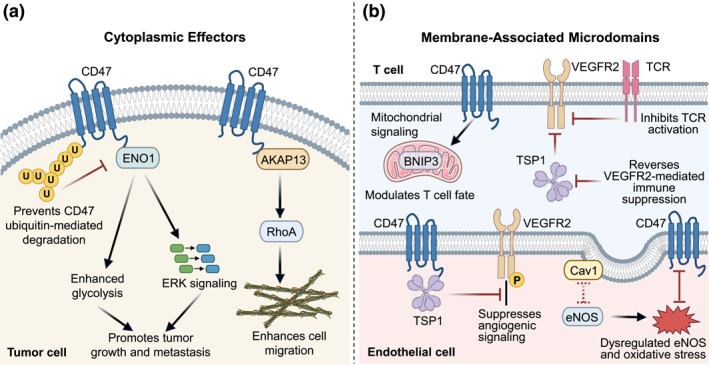
Intracellular signaling and membrane‐associated crosstalk of CD47. (a) Cytoplasmic effector signaling. CD47 engages intracellular effectors such as ENO1 and A‐kinase anchoring protein 13 to regulate receptor stability, glycolytic signaling, tumor growth, and cell migration. (b) Membrane‐associated signaling crosstalk. CD47 coordinates mitochondrial signaling and VEGFR2–TCR, TSP1–VEGFR2, and Cav1–eNOS signaling axes, thereby linking immune activation, angiogenesis, and vascular stress.

*Cytoplasmic binding proteins*: Accumulating evidence indicates that BCL2‐interacting protein 3 (BNIP3), α‐enolase (ENO1), and A‐kinase anchoring protein 13 (AKAP13) are representative intracellular effectors coupled with CD47, indicating that CD47 functions beyond immune checkpoints and engages intrinsic signaling pathways. In T cells, CD47 stimulation activates BNIP3‐associated mitochondrial pathways and influences cell fate, with distinct ligands eliciting divergent signaling outputs.^[46]^ In tumor cells, the interaction between CD47 and ENO1 protects CD47 from ubiquitin‐mediated degradation, enhances glycolysis and extracellular signal‐regulated kinase signaling, promotes tumor growth and metastasis, and indirectly shapes the immunosuppressive microenvironment.^[47]^ CD47 is also associated with AKAP13 to activate Ras homolog family member A (RhoA) and enhance tumor cell migration, supporting the existence of tumor‐intrinsic CD47 signaling pathways independent of phagocytosis inhibition^[48]^ (Figure 4a). These results demonstrate that CD47 exerts multilayered regulatory effects on intracellular signaling networks, providing mechanistic insights into tumor cell migration and metabolic adaptation while also revealing potential targets for early translational intervention.

*Membrane‐associated signaling*: CD47 integrates vascular and immune signaling pathways at the membrane level. In endothelial cells, TSP1–CD47 engagement suppresses VEGFR2 phosphorylation and downstream angiogenic signaling. This effect has been extensively validated in numerous experiments, highlighting the established mechanistic role of CD47 in angiogenic regulation.^[49]^ In T cells, VEGFR2 signaling inhibits TCR activation and proliferation, whereas TSP1 binding to CD47 or CD47 deletion partially reverses this suppression, indicating cross‐regulation between angiogenic and immune pathways.^[50]^ However, evidence for this mechanism is limited, primarily observed in cultured T cells and CD47 knockout mice, and it remains unclear whether this effect is overridden by immune checkpoints, such as PD‐1, in more complex microenvironments. In addition, CD47 upregulation promotes disease progression by limiting caveolin‐1 (Cav1)‐mediated inhibition of dysregulated endothelial nitric oxide synthase, thereby linking CD47 to oxidative stress and abnormal vascular tone^[51]^ (Figure 4b).

Collectively, CD47 forms a multidimensional interaction network with SIRPα, TSP1, and multiple integrins, positioning it as a central regulator of the interface between immune recognition and tissue homeostasis. Its core physiological roles include protecting normal cells through self‐recognition and suppression of macrophage phagocytosis, coordinating intercellular signaling during tissue development and repair, and maintaining immune homeostasis by limiting excessive immune activation.

## 4 PATHOLOGICAL ROLES OF CD47 IN DISEASE

CD47 is highly expressed in a wide range of solid tumors and hematological malignancies, and is consistently associated with poor prognosis, immune evasion, and aggressive phenotypes. Its pro‐tumorigenic effects arise from the combined actions of phagocytic checkpoint inhibition, tumor cell‐intrinsic pro‐invasive signaling, and the adaptive remodeling of metabolic and immune programs under therapeutic pressure.

### 4.1 Immune evasion

**FIGURE 5:**
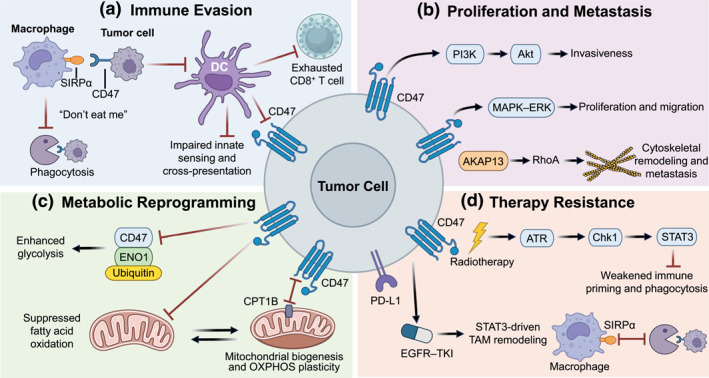
Multifaceted pathological mechanisms of CD47 in cancer. (a) Immune evasion. CD47–SIRPα signaling enables tumor cells to evade innate immune clearance and weakens downstream antitumor immunity. (b) Proliferation and metastasis. CD47 activates tumor‐intrinsic PI3K–Akt, MAPK–ERK, and AKAP13–RhoA signaling pathways, thereby promoting tumor growth, migration, and metastatic potential. (c) Metabolic reprogramming. CD47‐associated metabolic regulation enhances glycolysis, suppresses fatty acid oxidation, and supports mitochondrial and oxidative phosphorylation plasticity. (d) Therapy resistance. Treatment‐induced CD47 upregulation and STAT3‐associated tumor‐associated macrophage remodeling can weaken immune priming and reinforce resistance to radiotherapy or epidermal growth factor receptor–tyrosine kinase inhibitor therapy.

CD47 transmits a “don't eat me” signal through engagement with SIRPα on myeloid cells, thereby suppressing macrophage phagocytosis and promoting an M2‐like immunosuppressive phenotype in TAMs. Concurrently, this signaling axis reduces the expression of pro‐inflammatory cytokines and co‐stimulatory molecules in TAMs, thereby weakening innate immune sensing and DC‐mediated cross‐presentation, ultimately impairing T cell priming efficiency^[52]^ (Figure 5a). Within this suppressive microenvironment, TAMs further modulate the recruitment and function of neutrophils, fibroblasts, and effector T cells through inhibitory factors such as interleukin‐10 and transforming growth factor‐β, reinforcing the immunosuppressive network and restraining CD8^+^ T‐cell effector activity. Based on this mechanism, strategies that enhance tumor selectivity and combine CD47 blockade with other immunomodulatory approaches can amplify phagocytosis and the subsequent immune activation.^[53]^ In addition, CD47 signaling affects the functional state of T cells and contributes to immune escape. Elevated CD47 expression has been associated with exhaustion of tumor‐infiltrating CD8^+^ T cells.^[40]^ Collectively, the CD47 axis exerts dual regulation over myeloid cells and T cells, supporting the rationale for its targeting within mechanism‐driven combination strategies rather than as a standalone intervention.

### 4.2 Proliferation and metastasis

Beyond immune evasion, CD47 promotes tumor invasion and metastasis through tumor‐intrinsic signaling pathways. CD47 activation enhances glioblastoma invasiveness via phosphoinositide 3‐kinase–protein kinase B (PI3K–Akt) signaling.^[54]^ In craniopharyngiomas, CD47 suppresses microglial phagocytosis by activating mitogen‐activated protein kinase (MAPK)‐ERK signaling to promote proliferation and migration.^[55]^ In lymphoma models, CD47 coupling to AKAP13 activates RhoA and drives cytoskeletal remodeling, thereby increasing the metastatic potential^[48]^ (Figure 5b). These findings define a set of CD47‐dependent prometastatic mechanisms that are functionally separable from their antiphagocytic roles.

### 4.3 Metabolic reprogramming

CD47 interacts with ENO1 and prevents ubiquitin‐mediated degradation, resulting in enhanced glycolysis. Concurrently, CD47 suppresses fatty acid oxidation by downregulating carnitine palmitoyltransferase 1 B, collectively promoting tumor progression and metastasis. This metabolic axis has been reported in colorectal cancer and glioblastoma models[^56^, ^57^] (Figure 5c). Recent reviews further suggested that CD47 dynamically regulates mitochondrial biogenesis and oxidative phosphorylation, highlighting its pronounced metabolic plasticity.[^20^, ^58^] Although the current evidence is mainly derived from preclinical models, these studies suggest that CD47 may participate in a multilayered metabolic regulatory network. Further validation is needed to determine whether this metabolic axis can be therapeutically exploited across different tumor contexts.

### 4.4 Therapy resistance

Tumors with high CD47 expression can develop resistance by suppressing autophagy and inducing metabolic reprogramming in TAMs, thereby establishing a cooperative resistance niche between the tumor cells and myeloid microenvironment. In colorectal cancer, radiotherapy activates the ataxia telangiectasia and Rad3‐related protein–checkpoint kinase 1–signal transducer and activator of transcription 3 (ATR–Chk1–STAT3) pathway, leading to upregulation of CD47 and PD‐L1, suppression of antigen‐presenting cell phagocytosis, and tumor antigen cross‐presentation, ultimately weakening radiation‐induced immune priming^[59]^ (Figure 5d). Consistently, epidermal growth factor receptor–tyrosine kinase inhibitor resistance has been linked to STAT3‐driven TAM remodeling and enhanced CD47–SIRPα‐mediated inhibition of phagocytosis in non‐small cell lung cancer. Combined STAT3 inhibition and CD47 blockade partially reversed gefitinib resistance in this setting.^[60]^

## 5 CD47‐TARGETED CANCER IMMUNOTHERAPY: FROM FIRST‐GENERATION BLOCKADE TO SMART THERAPEUTIC SYSTEMS

CD47‐targeted cancer immunotherapy can be broadly categorized into three conceptual generations. First‐generation strategies use systemic CD47 or SIRPα blockade to establish therapeutic feasibility, while also revealing common limitations, such as antigen sink effects and hematologic toxicity. Second‐generation strategies use selectively optimized biologics to enhance intratumoral exposure or reduce non‐specific red blood cell (RBC) binding. Third‐generation approaches incorporate multifunctional, programmatically controlled systems for the precise spatiotemporal modulation of CD47 signaling. Importantly, this framework serves as a conceptual guide for therapeutic rationale and translational assessment, rather than an industry‐standard classification, strictly linear historical progression, or evidence that all third‐generation systems have achieved clinical maturity.

### 5.1 First‐generation CD47 blockade and translational bottlenecks

**FIGURE 6:**
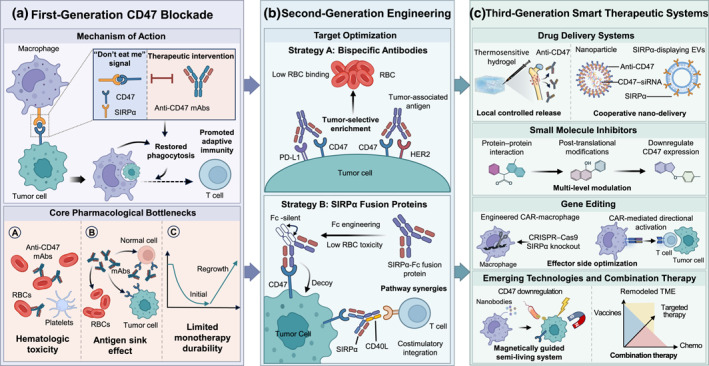
Evolution of CD47‐targeted cancer immunotherapy. (a) First‐generation CD47 blockade. Monoclonal antibodies disrupt CD47 signaling to restore macrophage phagocytosis, but systemic administration is associated with hematologic toxicity and antigen sink effects. (b) Second‐generation engineering. Bispecific antibodies and SIRPα fusion proteins improve tumor selectivity by reducing red blood cell engagement, enhancing tumor anchoring, and integrating additional immune‐activating signals. (c) Third‐generation smart therapeutic systems. Delivery platforms, small molecules, gene editing, nanobodies, physical guidance, and combination strategies aim to achieve spatially controlled, programmable, and multimodal regulation of the CD47 axis.

CD47‐targeted therapy has emerged from monoclonal antibodies, which established the CD47–SIRPα axis as a druggable myeloid checkpoint. By interrupting the “don't eat me” signal, these agents restore macrophage phagocytosis and promote adaptive antitumor immunity under appropriate conditions. However, first‐generation CD47 therapies have also exposed core pharmacological bottlenecks, such as hematologic toxicity, antigen sink, and limited durability of monotherapy. These limitations have motivated the development of more selective and engineered CD47‐targeted strategies (Figure 6a).

*Monoclonal antibodies*: Anti‐CD47 mAbs block the interaction between CD47 and SIRPα, thereby releasing phagocytic inhibition and enhancing macrophage‐mediated ADCP.^[2]^ Several anti‐CD47 mAbs have been clinically evaluated, with hematologic malignancies representing the earliest translational setting (Table 1). Magrolimab (Hu5F9‐G4), a first‐in‐class agent with a humanized immunoglobulin G4 (IgG4) backbone, restores macrophage phagocytic activity, while low‐dose priming mitigates anemia.^[12]^ Early phase combinations with rituximab or azacitidine showed preliminary efficacy in B‐cell lymphomas, myelodysplastic syndrome (MDS), and acute myeloid leukemia (AML); however, randomized trials have highlighted limitations imposed by the therapeutic window and hematologic safety.[^61^, ^62^] These limitations have led to the development of more differentiated designs. Therefore, current differentiation strategies focus on three directions: RBC–sparing antibodies such as AK117,^[63]^ IMC‐002,^[64]^ and STI‐6643^[65]^; TME–selective antibodies such as AO‐176^[66]^; and SIRPα‐side inhibitors such as BYON4228^[67]^ and DS‐1103a,^[68]^ which may bypass the RBC CD47 antigen sink and enhance ADCP when combined with tumor‐targeting antibodies or antibody‐drug conjugates. Despite these mechanistic refinements, most candidates remain in early clinical development, and whether they can achieve a stable, safe, and reproducible therapeutic window in patients remains unclear (Figure 6a).

**TABLE 1:** First‐generation CD47‐targeted immunotherapies in clinical development.

Drug name (code name)	Company name (sponsor)	Molecule format	Disease	Monotherapy or combination	Mechanism to reduce RBC binding	Main clinical advantage	Main limitation	Current development status	National clinical trial number (NCT no.)
Magrolimab (Hu5F9‐G4)	Gilead Sciences	Humanized IgG4 anti‐CD47 mAb	Solid tumors	Monotherapy	No RBC‐sparing design	Extensive clinical validation and strong macrophage activation	Significant anemia and antigen sink effect due to RBC binding	Phase I no longer recruiting; early solid‐tumor safety data reported	NCT02216409
			MDS; AML	Monotherapy				Phase I completed; hematologic malignancy data reported; no active recruitment	NCT02678338
			Hematological malignancies	Azacitidine				Phase Ib terminated; AML and MDS data reported	NCT03248479
			Non‐Hodgkin Lymphoma (NHL)	Acalabrutinib; ceralasertib; rituximab; AZD5153				Phase I platform study completed; no active recruitment	NCT03527147
			Lymphoma	Obinutuzumab; venetoclax				Phase I completed; no active recruitment	NCT04599634
			Brain tumors	Monotherapy				Phase I completed; brain‐tumor safety data limited; no active recruitment	NCT05169944
			MDS; AML	Sabatolimab; azacitidine				Phase I/II withdrawn; no patient enrollment or efficacy data reported	NCT05367401
	National Cancer Institute (NCI)	Humanized IgG4 anti‐CD47 mAb	Stage IV breast cancer; castration‐resistant prostate cancer	Olaparib	No RBC‐sparing design	Extensive clinical validation and strong macrophage activation	Significant anemia and antigen sink effect due to RBC binding	Phase I withdrawn; no patient‐dosing data reported	NCT05807126
	City of Hope Medical Center	Humanized IgG4 anti‐CD47 mAb	AML; MDS	Azacitidine	No RBC‐sparing design	Extensive clinical validation and strong macrophage activation	Significant anemia and antigen sink effect due to RBC binding	Phase I withdrawn; no patient‐dosing data reported	NCT05823480
CC‐90002 (not publicly specified)	Celgene	Humanized anti‐CD47 mAb; IgG subclass not consistently specified in public sources	Hematologic neoplasms	Monotherapy	No RBC‐sparing design	Early proof‐of‐concept for CD47 blockade	Limited efficacy and hematologic toxicity	Phase I completed; early safety data reported; no active development indicated	NCT02367196
			AML; MDS	Monotherapy				Phase I terminated during dose escalation; monotherapy data reported; no further development indicated	NCT02641002
Urabrelimab (SRF231)	Surface Oncology	Fully human IgG4 anti‐CD47 mAb	Solid tumors; hematological malignancies	Monotherapy	Partial affinity optimization	Enhanced macrophage‐mediated phagocytosis with favorable preclinical RBC‐safety profile	Limited mature efficacy data	Phase I platform study completed; no active recruitment	NCT03512340
Letaplimab (IBI188)	Innovent	Humanized IgG4 anti‐CD47 mAb	Advanced malignancies	Monotherapy	Affinity optimization	Reduced hematologic toxicity	Early‐stage validation only	Phase I completed; subsequent development appears limited	NCT03763149; NCT03717103
AO‐176 (not publicly specified)	Arch Oncology	Humanized IgG2 anti‐CD47 mAb	Solid tumors	Paclitaxel; pembrolizumab	Preferential tumor‐cell binding in acidic tumor microenvironment	Lower RBC binding and improved tumor selectivity	Limited large‐scale clinical evidence	Phase I/II completed; no active late‐stage development publicly reported	NCT03834948
			Multiple myeloma	Dexamethasone; dexamethasone—Bortezomib					NCT04445701
Gentulizumab (GenSci059)	GeneScience	Humanized IgG4 anti‐CD47 mAb	Solid tumors; NHL	Monotherapy	Affinity optimization	Favorable preclinical antitumor and safety profile	Limited publicly available efficacy data	Phase I terminated; limited public efficacy data available	NCT05221385
IMC‐002 (not publicly specified)	ImmuneOncia	Fully human IgG4 anti‐CD47 mAb	Advanced malignancies	Monotherapy	Fc engineering and affinity tuning	Improved safety profile	Limited efficacy data	Phase I active and recruiting; dose‐escalation and expansion data emerging	NCT05276310; NCT04306224
Ligufalimab (AK117)	Akeso	Humanized IgG4 anti‐CD47 mAb	MDS	Azacitidine	Epitope engineering	Lower anemia risk and broad combination potential	Mostly early clinical phases	Phase I/II ongoing	NCT04900350
			AML	Azacitidine				Phase I/II active, not recruiting	NCT04980885
			Advanced malignancies	Ivonescimab (AK112); chemotherapy				Phase I/II completed; solid‐tumor combination data reported	NCT05214482
			Advanced malignancies	AK112; carboplatin; cisplatin; 5‐fluorouracil (5‐FU)				Phase I/II active, not recruiting	NCT05229497
			Advanced malignancies	Cadonilimab (AK104); capecitabine; oxaliplatin; cisplatin; paclitaxel; irinotecan; docetaxel; 5‐FU				Phase I/II recruiting	NCT05235542
			Resectable gastric or gastroesophageal junction adenocarcinoma	Cadonilimab				Recruiting; phase II ongoing	NCT05960955
			AML	Azacitidine; venetoclax				Early phase I/II ongoing	NCT06387420
			Classic Hodgkin lymphoma	AK129				Recruiting; early phase I/II ongoing	NCT06642792
	West China Hospital	Humanized IgG4 anti‐CD47 mAb	Head and neck squamous cell carcinoma (HNSCC)	Anti‐EGFR antibody	Epitope engineering	Lower anemia risk and broad combination potential	Mostly early clinical phases	Phase II ongoing; trial‐specific status should be verified	NCT06508606
SHR‐1603‐based regimen (not publicly specified)	Chinese PLA General Hospital	Anti‐CD47 mAb; subtype details limited in public sources	Solid tumors	SHR2150; anti‐PD‐1	Not publicly specified	Explores multi‐axis innate and adaptive immune activation with CD47 blockade	Limited public drug‐level details	Phase I/II ongoing; combination study active; mature CD47‐specific data not reported	NCT04588324
STI‐6643 (not publicly specified)	Sorrento	Fully human IgG4 anti‐CD47 mAb	Solid tumors	Monotherapy	Epitope engineering	Improved safety window	Limited public clinical outcomes	Phase I recruiting; dose‐escalation study ongoing; mature efficacy data not reported	NCT04900519
HMPL‐A83 (not publicly specified)	Hutchmed	Humanized IgG4 anti‐CD47 mAb	Advanced tumors	Monotherapy	Affinity engineering	Reduced hematologic toxicity	Very early development	Phase I completed; no active recruitment	NCT05429008
BYON4228 (not publicly specified)	Byondis B.V.	Humanized anti‐SIRPα mAb	Lymphoma	Rituximab	Selective SIRPα blockade with no binding to RBC or platelets	Avoids hematologic toxicity and antigen sink effect	Clinical efficacy data still immature	Phase I active, not recruiting	NCT05737628
			Solid tumors	Pembrolizumab				Recruiting; phase I ongoing	NCT06932952
DS‐1103a (not publicly specified)	Daiichi Sankyo	Humanized IgG4 anti‐SIRPα mAb	Advanced solid tumors; breast cancer	Trastuzumab deruxtecan	Selective SIRPα blockade with no binding to RBC or platelets	Reduced anemia risk and favorable safety rationale	Limited published clinical efficacy results	Phase I ongoing	NCT05765851
Anti‐CD47 mAb (not publicly specified)	The First Affiliated Hospital of Soochow University	Anti‐CD47 mAb; subtype not publicly specified	Recurrent AML after transplantation	Azacitidine	Not publicly specified	Enhanced immune clearance potential with azacitidine in post‐transplant AML	Drug identity, subtype, RBC‐binding strategy, and efficacy data are limited publicly	Phase not applicable; status not clearly specified	NCT05266274
Anti‐SIRPα mAb (not publicly specified)	Nantes University Hospital	Anti‐SIRPα antibody; subtype not publicly specified	Hepatocellular carcinoma (HCC)	Monotherapy	Selective SIRPα blockade with no binding to RBC or platelets	Reduced RBC antigen sink and improved safety rationale	Drug identity and clinical efficacy data are limited in public sources	Phase not applicable; completed	NCT02868255

Abbreviation: RBC, red blood cell.

*Limitations of systemic CD47 blockade: Insights from magrolimab*: mAbs targeting the CD47–SIRPα axis provide a strong mechanistic rationale for restoring macrophage‐mediated phagocytosis. However, their clinical translation remains limited by hematological toxicity, antigen‐sink effects, and insufficient single‐agent activity. Magrolimab provides an illustrative example. Early single‐arm studies showed encouraging complete remission signals when magrolimab was combined with azacitidine in patients with MDS or AML, including those with TP53‐mutant disease.[^69^, ^70^] However, these findings were not confirmed in subsequent phase‐III randomized trials. This discrepancy suggests that small sample sizes, historical controls, and limited follow‐up may have overestimated early efficacy signals.

The ENHANCE trial failed to confirm the therapeutic advantages of magrolimab in MDS patients. ENHANCE‐II did not improve overall survival of patients with TP53‐mutant AML.^[71]^ ENHANCE‐III further showed that adding magrolimab to venetoclax plus azacitidine did not provide a survival benefit but was associated with increased infections, respiratory events, and anemia‐related toxicity.^[72]^ These results indicate that blocking the CD47‐mediated “don't eat me” signal alone is insufficient to overcome the genomic instability, therapeutic resistance, and suppressive myeloid microenvironment of TP53‐mutant disease. In the context of strong myelosuppressive combination regimens, systemic CD47 blockade may also amplify the safety risks.

Beyond magrolimab, the discontinuation or deprivation of other anti‐CD47 programs, such as gentulizumab, AO‐176, and urabrelimab, appears to have been influenced more by program‐specific factors, including competitive pressure, resource allocation, and corporate development strategy, than by safety or efficacy alone (Table 1). Nevertheless, these experiences highlight three common barriers to a systemic CD47 blockade. First, the broad expression of CD47 in RBCs, platelets, and normal tissues can limit tumor exposure and cause hematological toxicity. Second, the CD47 blockade alone may not provide sufficient “eat me” signals or antibody‐mediated opsonization. Third, combination regimens complicate the attribution of both efficacy and toxicity and still require validation in randomized studies.[^73^, ^74^, ^75^] Therefore, future strategies should emphasize mechanism‐matched combinations that expand the therapeutic window, improve effective tumor exposure, and enhance the reproducibility of clinical benefits.

### 5.2 Second‐generation engineering for selective targeting

First‐generation CD47 antibodies proved the pathway to be therapeutically tractable but also highlighted the limitations of systemic blockade. As a result, second‐generation development has moved toward a more refined pharmacological objective that retains myeloid checkpoint release while improving tumor selectivity and expanding the therapeutic window. This shift is most clearly reflected in target optimization strategies such as bispecific antibodies (BsAbs) and SIRPα‐based fusion proteins. These strategies can reduce RBC engagement and more precisely define the site, mechanism, and auxiliary functions of the CD47 blockade (Figure 6b). Here, we summarize second‐generation, highly selective CD47‐targeted immunotherapeutic strategies currently under clinical development (Table 2).

**TABLE 2:** Second‐generation highly selective CD47‐targeted immunotherapeutic strategies in clinical development.

Drug name (code name)	Company name (sponsor)	Molecule format	Disease	Monotherapy or combination	Mechanism to reduce RBC binding	Main clinical advantage	Main limitation	Current development status	National clinical trial
									Number (NCT no.)
CPO107 (not publicly specified)	Conjupro	Ofatumumab‐derived anti‐CD20 BsAb fused to SIRPα CD47‐binding domain	CD20‐positive NHL	Monotherapy	Tumor‐selective CD20 and SIRPα dual targeting	Tumor‐antigen anchoring may enhance selectivity and ADCP	Restricted to CD20‐positive tumors	Phase I/II no longer recruiting; mature efficacy data not yet reported	NCT04853329
PF‐07257876 (not publicly specified)	Pfizer	Fc‐active IgG1 CD47–PD‐L1 BsAb	Advanced solid tumors	Monotherapy	CD47–PD‐L1 dual targeting redistributes specificity	Simultaneous myeloid and T cell checkpoint modulation	Complex PK/PD profile	Phase I completed; early phase I data reported; no active recruitment	NCT04881045
HX009 (not publicly specified)	Waterstone Biopharma	Recombinant humanized PD‐1–CD47 BsAb	Advanced solid tumors	Monotherapy	CD47–PD‐1 dual targeting	Dual modulation of T cell and myeloid checkpoints	Limited efficacy maturity	Early phase I/II development	NCT04886271; NCT04097769
			Lymphoma					Phase II ongoing, not recruiting; MSS colorectal cancer data reported; dose optimization remains under evaluation	NCT05189093
IBC0966 (not publicly specified)	SunHo BioPharmaceutical	Not publicly specified	Advanced malignancies	Monotherapy	CD47–PD‐L1 selective targeting	Enhanced tumor immune specificity	Early clinical stage	Early phase I/II development	NCT04980690
Simridarlimab (IBI322)	Innovent Biologics	Recombinant anti‐CD47–PD‐L1 BsAb (Fc subclass not publicly specified)	Anti‐PD‐1 or anti‐PD‐L1 treatment‐resistant classical Hodgkin lymphoma (cHL)	Monotherapy	CD47–PD‐L1 selective targeting	Dual checkpoint modulation in resistant cHL	Early clinical stage	Phase I completed; dose‐expansion data reported in anti‐PD‐1 or anti‐PD‐L1‐resistant cHL	NCT04795128
NI‐1801 (not publicly specified)	Light Chain Bioscience	Mesothelin‐anchored CD47 BsAb	Epithelial ovarian cancer; triple‐negative breast cancer; non‐squamous non‐small cell lung cancer; pancreatic ductal adenocarcinoma (PDAC); endometrioid ovarian cancer	Single or combined with anti‐PD‐1 antibody; weekly paclitaxel (standard of care)	Mesothelin‐guided tumor targeting	Tumor‐restricted CD47 inhibition	Limited to mesothelin‐positive tumors	Recruiting; phase I ongoing	NCT05403554
Zeripatamig (TG‐1801)	TG Therapeutics	Fully human IgG1‐based anti‐CD19–anti‐CD47 BsAb	B‐cell lymphoma; chronic lymphocytic leukemia (CLL)	Ublituximab	CD19 anchoring localizes CD47 blockade to CD19+ B cells	Enhances CD19‐directed phagocytic tumor clearance	Limited indications for tumor treatment	Phase Ib terminated; ublituximab combination discontinued; mature efficacy data not reported	NCT04806035
IMM2520 (not publicly specified)	ImmuneOnco Biopharmaceuticals	IgG1 Fc‐containing PD‐L1 antibody linked to the SIRPα domain	Advanced solid tumors; non‐small cell lung cancer; small cell lung cancer; breast cancer; squamous cell cancer of head and neck; colorectal cancer	Monotherapy	PD‐L1‐anchored SIRPα‐trap format localizes CD47 blockade to PD‐L1‐expressing tumors	Integrates PD‐L1 blockade, CD47–SIRPα blockade, ADCP and Fc effector functions	Insufficient public mechanistic and efficacy data	Recruiting; phase I ongoing	NCT05780307
Vislarafusp alfa (IMM2902)	ImmuneOnco Biopharmaceuticals (Shanghai) Inc.	HER2‐targeted SIRPα‐trap BsAb; Fc subclass not publicly specified	HER2‐expressing advanced solid tumors	Monotherapy	HER2 anchoring localizes CD47 blockade to HER2‐expressing tumors	HER2‐guided phagocytic activation with reduced antigen sink rationale	Early‐phase evidence	Phase I/II recruiting; mature efficacy data not reported	NCT05805956
Peluntamig (PT217)	Phanes Therapeutics	IgG1‐based anti‐DLL3; anti‐CD47 BsAb	Small cell lung cancer (SCLC); large cell neuroendocrine cancer (LCNEC); neuroendocrine prostate cancer (NEPC); gastroenteropancreatic neuroendocrine carcinoma (GEP‐NEC); neuroendocrine carcinomas (NEC); extrapulmonary neuroendocrine carcinoma (EP‐NEC)	Carboplatin–etoposide; paclitaxel; atezolizumab	CD47–DLL3 dual specificity	Neuroendocrine tumor specificity	Narrow indication scope (restricted to DLL3‐expressing tumors)	Recruiting; phase I/II ongoing	NCT05652686
AK132 (not publicly specified)	Akeso	CLDN18.2–CD47 BsAb with IgG1 Fc	Advanced malignant solid tumors	Monotherapy	CLDN18.2‐anchored format localizes CD47 blockade to tumor cells	Potentially combines tumor anchoring, CD47 blockade, and Fc effector function	Limited public clinical efficacy data and biomarker dependence on CLDN18.2 expression	Early phase I development	NCT06166472
Ontorpacept (TTI‐621)	Trillium Therapeutics	SIRPα‐IgG1 Fc fusion protein	Relapsed or refractory hematologic malignancies; selected solid tumors	Rituximab; nivolumab	SIRPα‐decoy binding to CD47 avoids an anti‐CD47 Fab format	Early clinical validation of SIRPα‐Fc blockade and macrophage activation	Thrombocytopenia risk and limited durable monotherapy efficacy	Phase I administratively complete; no active development indicated	NCT02663518
			Relapsed or refractory cutaneous T‐cell lymphoma; selected solid tumors	PD‐1 or PD‐L1 inhibitor; pegylated interferon alpha‐2a; talimogene laherparepvec				Phase I sponsor‐closed; intralesional data reported; no longer recruiting	NCT02890368
Evorpacept (ALX148)	ALX Oncology	High‐affinity SIRPα D1 domain fused to inactive IgG1 Fc	Advanced solid tumors; NHL	Pembrolizumab; trastuzumab; rituximab; ramucirumab–paclitaxel; 5‐FU–cisplatin	Engineered inactive Fc; high‐affinity SIRPα domain	Minimal hematologic toxicity and broad combinability	Limited standalone efficacy; benefit is combination‐dependent	Phase I completed; early data reported; no longer recruiting	NCT03013218
			HER2‐overexpressing gastric or gastroesophageal junction adenocarcinoma	Trastuzumab; ramucirumab; paclitaxel				Phase II/III program ongoing; phase II data reported	NCT05002127
			Microsatellite‐stable metastatic colorectal cancer	Cetuximab; pembrolizumab				Phase I active, not recruiting	NCT05167409
			MDS	Azacitidine				Phase I/II no longer active; early MDS data reported; no ongoing recruitment	NCT04417517
			Head and neck cancer	Pembrolizumab			Did not meet primary response expectations in reported phase II HNSCC programs	Phase II active, not recruiting	NCT04675294
			Head and neck cancer	Pembrolizumab; cisplatin; carboplatin; 5‐FU			Did not meet primary response expectations in reported phase II HNSCC programs	Phase II active, not recruiting	NCT04675333
			AML	Venetoclax; azacitidine			Myelosuppressive backbone increases safety risk and confounds efficacy attribution	Phase I/II terminated; phase I AML data reported; no ongoing recruitment	NCT04755244
	Investigator initiated	High‐affinity SIRPα D1 domain fused to inactive IgG1 Fc	Platinum‐resistant ovarian cancer	Pembrolizumab; pegylated liposomal doxorubicin	Engineered inactive Fc; high‐affinity SIRPα domain	Minimal hematologic toxicity and broad combinability	Limited standalone efficacy; benefit is combination‐dependent	Phase II ongoing; not recruiting	NCT05467670
	Sanofi	High‐affinity SIRPα D1 domain fused to inactive IgG1 Fc	Relapsed or refractory multiple myeloma	Isatuximab; dexamethasone; pomalidomide; belantamab mafodotin; pegenzileukin; SAR439459; belumosudil; evorpacept	Engineered inactive Fc; high‐affinity SIRPα domain	Minimal hematologic toxicity and broad combinability	Limited standalone efficacy; benefit is combination‐dependent	Phase I–II recruiting; combination arm ongoing	NCT04643002
Maplirpacept (TTI‐622)	Trillium Therapeutics	SIRPα‐IgG4 Fc fusion protein	Platinum‐resistant recurrent epithelial ovarian cancer	Pegylated liposomal doxorubicin	SIRPα‐IgG4 Fc format minimally binds human RBCs	Reduced hematologic toxicity with broad combinability	Potential thrombocytopenia and limited monotherapy efficacy	Phase I/II terminated; not safety‐related	NCT05261490
		SIRPα‐IgG4 Fc fusion protein	Relapsed or refractory multiple myeloma	Daratumumab hyaluronidase‐fihj	SIRPα‐decoy binding to CD47 avoids an anti‐CD47 Fab format	Early clinical validation of SIRPα‐Fc blockade and macrophage activation	Thrombocytopenia risk and limited durable monotherapy efficacy	Phase II/III active, not recruiting	NCT05139225
	Pfizer	SIRPα‐IgG4 Fc fusion protein	Multiple myeloma	Elranatamab; carfilzomib	SIRPα‐IgG4 Fc format minimally binds human RBCs	Supports combination with B‐cell maturation antigen (BCMA)‐targeted and proteasome‐inhibitor backbones	May still cause thrombocytopenia	Recruiting; phase I ongoing	NCT05675449
			Diffuse large B‐cell lymphoma	Glofitamab; obinutuzumab		Strengthens B cell‐directed immunotherapy through CD47 blockade		Recruiting; phase I/II ongoing	NCT05896163
			Diffuse Large B‐cell lymphoma	Tafasitamab; lenalidomide	SIRPα‐decoy binding to CD47 avoids an anti‐CD47 Fab format	Promotes CD19‐directed opsonization and macrophage‐mediated tumor clearance		Recruiting; phase I/II ongoing	NCT05626322
HCB101 (not publicly specified)	FBD Biologics Limited	Engineered human SIRPα extracellular domain fused to human IgG4 Fc	Advanced malignancies	Monotherapy	Engineered SIRPα fusion protein with reduced hematologic binding profile	Lower RBC binding and improved safety profile	Early‐stage development with limited efficacy validation	Recruiting; phase I ongoing	NCT05892718
SL‐172154 (not publicly specified)	Shattuck Labs	Hexameric SIRPα‐Fc‐CD40L fusion protein with inert IgG4‐derived Fc	Platinum‐resistant ovarian cancer	Monotherapy	Inert Fc reduces Fc‐mediated blood‐cell clearance	Combines CD47 blockade with CD40 costimulation	Limited objective responses	Phase I completed; first‐in‐human monotherapy data reported	NCT04406623

Abbreviations: MSS, microsatellite stable; RBC, red blood cell.

*Bispecific antibodies*: Current clinical bispecific approaches fall into two categories: immune checkpoint co‐targeting and tumor antigen‐anchored design. The former aims to achieve complementary regulation across multiple immune pathways. For example, IBI322 (CD47–PD‐L1) simultaneously relieves myeloid phagocytic suppression and PD‐1–PD‐L1‐mediated T cell inhibition, with phase I data in classical Hodgkin lymphoma refractory to prior PD‐1–PD‐L1 therapy showing acceptable safety and preliminary efficacy.^[76]^ The latter spatially restricts the CD47 blockade in malignant cells. Zeripatamig (TG1801, CD19–CD47) has demonstrated early safety and efficacy in CD19^+^ B‐cell non‐Hodgkin lymphoma (NHL), although its activity in CD19‐low tumors remains to be validated.^[77]^ Similarly, Vislarafusp alfa (IMM2902, HER2–CD47) leverages human epidermal growth factor receptor 2‐mediated internalization to enhance tumor accumulation and phagocytosis while limiting systemic exposure, although its clinical development has been paused for strategic reasons.^[78]^ These cases highlight the shift from systemic CD47 blockade toward tumor‐anchored, intramolecularly synergistic designs with potential in solid tumors, although their clinical impact still requires more mature efficacy data, survival endpoints, and biomarker‐guided randomized evaluations.

*SIRPα fusion proteins*: SIRPα‐based fusion proteins provide a complementary receptor‐side strategy using SIRPα domains as CD47 decoys, with Fc engineering to reduce RBC engagement and hematologic toxicity. Conventional SIRPα‐Fc proteins, such as HCB101, fuse engineered SIRPα ECD to IgG4 Fc, achieving high CD47 affinity. Phase I data show favorable disease control and tolerability in advanced gastric cancer and NHL, although randomized survival endpoints are lacking, and single‐agent efficacy remains limited.^[79]^ Notably, Fc‐silent designs, exemplified by Evorpacept (ALX148), combine high‐affinity SIRPα D1 with inactivated Fc to block CD47–SIRPα signaling, while minimizing Fcγ receptor activation and nonspecific blood cell clearance. Early clinical studies suggested favorable tolerability and combination activity in HER2‐positive gastric cancer, Head and neck squamous cell carcinoma, and refractory high‐risk NHL.[^80^, ^81^, ^82^] Beyond decoy blockade, next‐generation fusion proteins integrate CD47 inhibition with immune activation, as illustrated by SL‐172154, which combines SIRPα‐Fc with the CD40 ligand to relieve phagocytic inhibition while activating antigen‐presenting cells and T cell pathways.^[83]^

*Target optimization and clinical translational assessment*: Optimization of CD47‐targeted therapeutics aims to reconcile potent myeloid checkpoint blockade with hematologic safety. Current strategies focus on enhancing tumor‐specific accumulation or implementing receptor‐side interventions targeting SIRPα to mitigate RBC engagement and related toxicity. BsAbs achieve intratumoral enrichment through tumor antigen anchoring, whereas receptor‐side designs circumvent the direct CD47 engagement. Nonetheless, second‐generation therapeutics face translational hurdles: early phase clinical evidence remains limited, antigen sink and rapid clearance may compromise exposure, and structural complexity poses manufacturing and reproducibility challenges.[^84^, ^85^] Predictive biomarkers are scarce, but patient stratification integrating co‐expression of tumor antigens (e.g., PD‐L1–CD47 and MSLN–CD47), immunosuppressive microenvironmental features, and auxiliary indicators such as tumor mutational burden could refine therapeutic selection.^[86]^ Collectively, these strategies may expand the therapeutic window of CD47‐targeted immunotherapy; however, their clinical value will require randomized validation and more precise biomarker‐guided development.

### 5.3 Third‐generation smart therapeutic systems

Third‐generation CD47‐targeted therapy is no longer confined to safer checkpoint blockade but has expanded to spatiotemporal control, conditional activation, programmable multifunctionality, and pathway‐level rewiring. At this stage, CD47‐directed therapy began to operate less as a single‐axis blocking strategy and more as an intelligent therapeutic platform (Figure 6c). Here, we summarize the representative third‐generation smart immunotherapeutic strategies targeting CD47 that have emerged in recent years (Table 3).

**TABLE 3:** Representative third‐generation smart immunotherapeutic strategies targeting CD47.

Smart therapeutic system category	System name	Target	Disease	Functional component(s)	Monotherapy or combination therapy	Main effect(s)
Drug delivery system	LPH(CD47) NP^[87]^	CD47	Melanoma	CD47 siRNA	Monotherapy	CD47 gene silencing
Drug delivery system	SNPA CALR&aCD47^[88]^	CD47; calreticulin	Breast cancer	Anti‐CD47 antibody (aCD47) and calreticulin	Combination therapy	Blocks the CD47–SIRPα axis
Drug delivery system	DLG hierarchical gel^[89]^	CD47–SIRPα	Breast cancer	aCD47 and sorafenib	Combination therapy	Remodels TAMs and continuously blocking CD47
Drug delivery system	hGLV exosome‐liposome hybrid nanovesicles^[90]^	CD47	Colon cancer	Indocyanine green (ICG) and imiquimod (R837)	Combination therapy	Blocks the CD47–SIRPα axis
Drug delivery system	PQ/PB‐Gel injectable hydrogel^[91]^	QPCTL‐mediated CD47 pGlu formation	Melanoma; breast cancer	PQ912	Monotherapy	Attenuates functional CD47–SIRPα recognition
Drug delivery system	OAd‐mCD47nb‐Fc^[92]^	CD47	Melanoma; lymphoma; breast cancer	aCD47‐Fc expressed by oncolytic adenovirus	Combination therapy	Local blockade of CD47
Drug delivery system	aCD47‐DMSN^[93]^	CD47	Melanoma; breast cancer	aCD47 and doxorubicin	Combination therapy	Enhances chemotherapy‐associated phagocytosis
Drug delivery system	NP‐R848‐siCD47^[94]^	CD47; toll‐like receptor 7/8 (TLR7/8)	Breast cancer	CD47 siRNA and resiquimod (R848)	Combination therapy	CD47 gene silencing
Drug delivery system	SPI@hEL‐RS17^[95]^	CD47	Melanoma; breast cancer	Shikonin, IR820, polymetformin and RS17 peptide	Combination therapy	Blocks the CD47–SIRPα axis
Drug delivery system	SIRPα@Zeb nanovesicles^[96]^	CD47–SIRPα	Melanoma	SIRPα‐displaying nanovesicles and zebularine	Combination therapy	Blocks SIRPα and promote M1 polarization
Drug delivery system	OVV‐αCD47nb^[97]^	CD47	Breast cancer; colon cancer	aCD47 expressed by oncolytic vaccinia virus	Combination therapy	Blocks the CD47–SIRPα axis
Drug delivery system	MDCPA/aCD47 thermo‐sensitive injectable hydrogel^[98]^	CD47	Prostate cancer bone metastasis	MDCPA and aCD47	Combination therapy	Remodels TME and continuously blocking CD47
Drug delivery system	Anti‐CD47–anti‐PD‐L1 ADN^[99]^	CD47; PD‐L1; PI3K	NSCLC	aCD47, anti‐PD‐L1 antibody and PI103	Combination therapy	Remodels CD47 and PD‐L1
Small molecule inhibitor	SMC18^[100]^	CD47–SIRPα; PD‐1–PD‐L1	Colorectal cancer	—	Monotherapy	Remodels CD47 and PD‐L1
Small molecule inhibitor	22b^[101]^	Glutaminyl cyclase (QC)/isoQC	Lung cancer	—	Monotherapy	Blocks the CD47–SIRPα axis
Small molecule inhibitor	QP5038^[102]^	QPCTL	Lymphoma; melanoma	QPCTL inhibitor	Combination therapy	Blocks the CD47–SIRPα axis
Small molecule inhibitor	Compound 30^[103]^	Golgi‐resident glutaminyl cyclase (gQC) and secreted glutaminyl cyclase (sQC)	Breast cancer	Glutaminyl cyclase inhibitor with anti‐PD‐1	Combination therapy	Blocks the CD47–SIRPα axis
Small molecule inhibitor	RRx‐001^[104]^	CD47–SIRPα signaling	Breast cancer; lung cancer	—	Monotherapy	Downregulates CD47–SIRPα signaling
Small molecule inhibitor	ML364^[32]^	USP2	Lung cancer	USP2 inhibitor with anti‐PD‐1	Combination therapy	Reduces CD47 stability and enhances phagocytosis
Small molecule inhibitor	Luteolin^[105]^	isoQC	Myeloma; colorectal cancer	—	Monotherapy	Blocks the CD47–SIRPα axis
Genetic engineering	CD47 CAR‐M^[106]^	CD47	Ovarian cancer	—	Monotherapy	Antigen‐specific phagocytosis; TME reprogramming
Genetic engineering	HER2 CAR‐M^[107]^	HER2; SIRPα	Ovarian cancer; melanoma	—	Monotherapy	Enhances phagocytosis; reshape TME; activate cGAS‐STING
Genetic engineering	Anti‐CD47 CAR‐M(IL‐21)^[108]^	CD47	Ovarian cancer	—	Monotherapy	Enhances phagocytosis; promotes M1 polarization and cytotoxic T lymphocyte activation
Genetic engineering	pArg1‐CD47 CAR‐Mφ^[109]^	CD47	Ovarian cancer; gastric cancer	—	Monotherapy	Enhances phagocytosis; reshape TME
Genetic engineering	eSPR macrophage^[110]^	HER2	Metastatic solid tumors with antigen heterogeneity	—	Monotherapy	Overcomes antigen heterogeneity; promotes cross‐presentation
Genetic engineering	Sirf CAR‐T^[111]^	CD47; Trop2	NSCLC; breast cancer	—	Monotherapy	Enhances phagocytosis; recruits DCs and T cells
Genetic engineering	Orexi CAR‐T^[112]^	CD19; CD47	B‐cell tumors	Rituximab	Combination therapy	Enhances antibody‐dependent killing
Genetic engineering	47E protective CAR/TCR‐T^[113]^	CD47 (Q31P)‐47E; SIRPα	Osteosarcoma; melanoma; neuroblastoma	aCD47	Combination therapy	Enhances adaptive immunity
Genetic engineering	CV1‐Decoy engineered TCR‐T^[114]^	CD47	Melanoma	Cetuximab; avelumab	Combination therapy	Enhances phagocytosis
Genetic engineering	Tumor‐associated mucin 1 (tMUC1) CAR‐M^[115]^	SIRPα; tMUC1	Leukemia; breast cancer	—	Monotherapy	Enhances phagocytosis
Genetic engineering	CV1‐CAR T^[116]^	CD47 ephrin type‐A receptor 2 (EphA2); CD47–SIRPα	NSCLC	—	Monotherapy	Enhances adaptive immunity
Physical therapy	Photothermal therapy^[117]^	CD47	Breast cancer	aCD47‐modified Bi_2_Se_3_ nanoparticle (NP)	Combination therapy	Enhances phagocytosis
Physical therapy	Photothermal therapy^[118]^	CD47	Breast cancer	IR820–aCD47	Combination therapy	Blocks the CD47–SIRPα axis
Physical therapy	Photodynamic therapy^[119]^	CD47	Osteosarcoma	Chlorin e6 (Ce6) and aCD47	Combination therapy	Enhances phagocytosis
Physical therapy	Photothermal therapy^[120]^	CD47	Oral squamous cell carcinoma	aCD47	Combination therapy	Enhances phagocytosis and adaptive immunity
Physical therapy	Photothermal therapy^[121]^	CD47	Glioblastoma	A1094 dye, temozolomide (TMZ), and aCD47	Combination therapy	Enhances phagocytosis; reshapes TME
Physical therapy	Sonodynamic therapy^[122]^	CD47	Osteosarcoma	IR780, RRx‐001, and MPIRx	Combination therapy	Enhances phagocytosis and adaptive immunity
Physical therapy	Sonodynamic therapy^[123]^	CD47	Breast cancer	IR780 and MnO_2_@PLGA NPs	Combination therapy	Enhances phagocytosis and adaptive immunity
Physical therapy	Focused ultrasound therapy^[124]^	SIRPα	NSCLC	SIRPα siRNA, Fe_3_O_4_ and folic acid‐modified NPs	Combination therapy	Inhibits SIRPα; enhances phagocytosis
Physical therapy	Focused ultrasound therapy^[125]^	CD47	Glioma	aCD47	Combination therapy	Enhances phagocytosis
Physical therapy	Magnetothermal therapy^[126]^	CD47–SIRPα	HCC	Ferrimagnetic vortex‐domain iron oxide (FVIO) nanorings	Monotherapy	Enhances phagocytosis; reshape TME
Physical therapy	Photodynamic therapy^[127]^	CD47	Breast cancer	Iridium(III) complex	Combination therapy	Reshapes TME

Abbreviations: MDCPA, biomimetic mineralized dicalcium phosphate anhydrous; PLGA, poly (lactic‐co‐glycolic acid).

*Drug delivery systems*: Delivery strategies aim to reduce systemic exposure, increase intratumoral drug concentrations, and enable controlled drug release. For local lesions, thermosensitive injectable hydrogels loaded with biomimetic mineralized dicalcium phosphate anhydrous and anti‐CD47 antibodies can solidify in situ at bone metastases, continuously release drugs, remodel the bone microenvironment, and enhance phagocytosis, thereby suppressing prostate cancer bone metastasis.^[98]^ In systemic settings, nanocarriers enable a coordinated immune modulation. For example, antibody‐drug‐nanoparticle conjugates (ADNs) co‐delivering anti‐CD47 and anti‐PD‐L1 with the PI3K inhibitor PI103 achieve tumor enrichment and concurrently relieve innate and adaptive immune suppression.^[99]^ This design enabled the integrated delivery of multiple immunomodulatory signals. However, its efficacy depends on the delivery efficiency, release kinetics, and synchronized tumor enrichment, making reproducibility and in vivo pharmacokinetics critical challenges. Recently, cell‐engineered vesicles have emerged as a promising platform for CD47‐targeted delivery. SIRPα‐displaying EVs developed by Nam et al. block the CD47 axis, reduce hematologic toxicity, and enhance phagocytosis and immune activation.^[128]^ These vesicles offer the advantages of crossing biological barriers, improving tissue distribution, and achieving biocompatible delivery, particularly for solid or brain tumors with limited antibody exposure. Therefore, local hydrogels and engineered vesicles represent near‐term translational opportunities, whereas highly complex multicomponent nanocarriers require clear efficacy advantages, scalable manufacturing, and regulatory feasibility to support clinical advancement.

*Small‐molecule inhibitors*: Small‐molecule strategies targeting the CD47 axis have expanded from direct disruption of protein‐protein interactions to multi‐level modulation aimed at improving tumor selectivity and safety. Zhao et al. reported that SMC18 simultaneously interferes with SIRPα and PD‐L1 interactions and promotes phagocytosis and immune activation through dual pathways, providing proof‐of‐concept evidence for small‐molecule coordination of myeloid and T cell checkpoints.^[100]^ However, the flat and extensive CD47–SIRPα interface limits the achievable affinity of conventional small molecules, which has motivated the development of medium‐sized ligands. Gao et al. identified the peptide pep‐20 from phage‐display libraries, which binds human and murine CD47 with micromolar affinity comparable to that of native SIRPα and blocks CD47–SIRPα interactions in a dose‐dependent manner.^[129]^ Targeting of PTMs is an alternative approach. Benzimidazole‐based glutaminyl cyclase (QC) inhibitors prevent N‐terminal pGlu formation on CD47, reduce SIRPα binding, and enhance phagocytosis.^[103]^ In addition, small molecules such as RRx‐001 have been reported to downregulate CD47 and SIRPα expression, reshape phagocytic thresholds at the source, provide complementary strategies to reduce systemic toxicity, and support combination regimens.^[104]^ Overall, small‐molecule‐ and peptide‐based strategies offer advantages in terms of manufacturability and tissue penetration. However, clinically validated alternatives have not been developed yet. Their greatest potential may lie in their use as adjuncts to antibody‐ or cell‐based therapies, where they can modulate CD47 function, PTMs, or the TME.

*Gene editing*: Gene‐editing approaches reshape phagocytic thresholds from the effector cell side. In 2025, Wattanapanitch et al. showed that SIRPα knockout enhances macrophage phagocytosis of tumor cells. However, in solid tumors, this effect requires coupling with chimeric antigen receptor‐mediated targeting to achieve sufficient amplification, indicating that the release of inhibition must be paired with directional activation.^[115]^ In CAR‐macrophage systems, SIRPα silencing enhances antitumor efficacy in solid tumors, demonstrating that receptor‐side inhibition combined with engineered recognition markedly strengthens phagocytosis.^[107]^ Importantly, CD47‐axis interventions may antagonize T cell therapies. CD47 expression has been shown to be critical for CAR‐T cell persistence in vivo, suggesting that CD47 blockade may compromise transferred cell survival.^[116]^ To address this, engineered CD47 variants that protect T cells from anti‐CD47 recognition have been developed to restore and enhance the combinatorial antitumor activity.^[113]^ These findings indicate that CD47‐targeted agents and cell therapies require a systematic co‐design rather than a simple combination. These technologies remain at an early translational stage, with unresolved challenges in terms of quality control, trigger delivery, safety, and regulatory approval. Nevertheless, these results may provide useful design principles for next‐generation CD47‐targeted therapeutics.

*Emerging technologies*: New CD47 immunotherapy technologies integrate innate and adaptive immune amplification, microenvironment remodeling, and spatiotemporal control to address hematologic toxicity, insufficient immune activation, cold tumors, and unintended immune cell clearance. Jiang et al. developed a CD47 antibody‐listeriolysin O conjugate that perforates phagosomal membranes after tumor cell uptake, facilitating cytosolic antigen delivery, cross‐presentation, and innate sensing, thereby amplifying systemic T‐cell responses.^[130]^ The integration of physical modalities provides an additional avenue. Photoresponsive Ir(III) complexes induce immunogenic cell death and downregulate CD47 through reactive oxygen species generation, strengthening “eat‐me” signals at the source.^[127]^ Magnetically responsive strategies enable spatial control. Engineered *Escherichia coli* carrying anti‐CD47 antibodies, combined with phase‐change materials and ferrofluid isolation, form magnetically guided “semi‐living” systems that target tumors, while protecting peritumoral effector T cells and mitigating T cell loss associated with CD47 blockade.^[131]^ Collectively, these emerging technologies integrate immune enhancement with spatial and temporal control, thereby offering innovative solutions for overcoming the current limitations of CD47‐based immunotherapy. Nevertheless, these technologies remain far from clinical translation and face challenges beyond efficacy, including long‐term safety concerns, limited in vivo trigger accessibility, and unclear regulatory mechanisms. Currently, they are better suited for mechanistic exploration and platform innovation than mature clinical alternatives.

*Combination therapy*: Although CD47 blockade releases phagocytic inhibition, durable clinical benefit relies on adequate “eat‐me” signal supply and immune amplification. Combining CD47 inhibitors with chemotherapy leverages immunogenic cell death and exposure to prophagocytic signals, as illustrated by evorpacept plus paclitaxel in HER2‐positive gastric cancer, which integrates standard chemotherapy with enhanced phagocytosis.^[132]^ Combinations with targeted therapies emphasize the dual‐layer synergy. PPAB001, a CD47–CD24 bispecific antibody, reprograms TAMs toward the M1 phenotype and enhances anti‐PD‐L1 efficacy in triple‐negative breast cancer.^[133]^ When combined with vaccines, intracellularly gelled tumor vaccines markedly enhance immune recognition in the presence of CD47 blockade and damage‐associated molecular pattern exposure.^[134]^ In lymphoma models, oncolytic vaccinia virus carrying anti‐CD47 nanobodies synergizes with CD19 CAR‐T cells, enhancing innate immune activation while preventing unintended clearance of effector cells.^[135]^ Translationally, CD47 combination therapies can practically compensate for limited “eat‐me” signals and insufficient immune amplification of single‐agent blockade but carry risks of cumulative toxicity, unclear sequencing, and complex patient selection. Future strategies should avoid indiscriminate addition and should instead be mechanistically matched to tumor antigens, pro‐phagocytic signals, TAM states, and hematologic tolerance.

### 5.4 CD47 therapeutics: Mechanistic evolution and biomarker‐guided precision

Despite substantial translational challenges, CD47 remains a highly relevant immunotherapy target. Recent clinical setbacks have defined the limitations of systemic CD47 blockade rather than undermining the biological importance of the CD47–SIRPα axis. Future progress will depend on tumor‐selective exposure, rational combination strategies, biomarker‐guided patient selection, and controlled therapeutic deployment. In this context, a clinically actionable biomarker framework is essential for precise intervention and patient stratification.

Such a framework should integrate tumor‐intrinsic target features with the functional state of the phagocytic immune cells. Key parameters include tumor CD47 abundance, expression of bispecific antibody co‐targets, infiltration of SIRPα‐positive TAMs, density of FcγR‐competent macrophages and DCs, pro‐phagocytic signals such as calreticulin, macrophage functional status, tumor antigenicity, additional anti‐phagocytic checkpoints such as CD24–Siglec‐10, and baseline hematopoietic reserve.[^136^, ^137^] For PTM‐targeted strategies, N‐terminal pyroglutamylation mediated by QPCTL and CD47 glycosylation patterns may serve as informative indicators.[^30^, ^31^] Integrating these metrics may help to guide combination therapy design, patient selection, and clinical translation with greater precision.

## 6 CONCLUSION

The CD47–SIRPα axis represents a central phagocytic checkpoint and provides a therapeutic entry point distinct from the PD‐1–PD‐L1 pathway for reprogramming myeloid cell‐mediated antitumor immunity. Its value lies not only in releasing macrophage phagocytic inhibition but also in enhancing antigen uptake and cross‐presentation, thereby enabling a cascade from innate to adaptive immune activation and offering new translational opportunities for immunologically “cold” tumors.^[4]^ However, repeated setbacks in clinical development indicate that the success of CD47‐targeted strategies ultimately depends on achieving a reproducible engineering balance between efficacy, hematologic safety, and effective tumor exposure.^[11]^

Future breakthroughs will likely rely on integrated context‐aware approaches. Conditional activation, TME‐selective targeting, and molecular engineering are required to reduce peripheral blood cell engagement and narrow the systemic toxicity window. In contrast, bispecific formats, advanced delivery systems, and ligand‐ or receptor‐side targeting strategies can improve intratumoral drug concentration and selective exposure. Importantly, the therapeutic value of the CD47 blockade should be redefined within combination paradigms rather than as monotherapy, with particular promise for combinations with immune checkpoint inhibitors.^[138]^ Small‐molecule approaches offer additional advantages in oral availability, controllable exposure, and combinatorial flexibility and may enable the coordinated release of innate and adaptive immune inhibition while reducing hematologic risk and improving the reproducibility and scalability of clinical benefits.[^14^, ^139^]

Overall, CD47 has emerged as a bona fide phagocytic checkpoint that expands the conceptual and therapeutic landscape of cancer immunotherapy, while simultaneously introducing new challenges. Several key questions remain unresolved: how to fundamentally overcome the structural trade‐off between antigen sink effects and hematologic toxicity, which druggable nodes beyond QPCTL exist within CD47 PTM networks, how tumors acquire resistance following CD47 blockade, whether alternative antiphagocytic pathways should be prioritized in combination strategies, and when the first CD47–SIRPα‐targeted therapy will achieve regulatory approval. As these questions are addressed, clearer frameworks are expected to emerge for defining therapeutic windows, exposure boundaries, responsive patient populations, and rational combination strategies, ultimately advancing CD47‐targeted therapies toward a more mature and effective treatment paradigm.

## AUTHOR CONTRIBUTIONS

**Ruimei Zhou**: Methodology; software; formal analysis; investigation; visualization; data curation; writing—original draft; writing—review and editing. **Lingjie Jing**: Methodology; software; writing—original draft; writing—review and editing. **Jianping Zhang**: Visualization; investigation; writing—original draft; writing—review and editing. **Cheng Guo**: Supervision; resources; validation; writing—original draft; writing—review and editing. **Quanjun Yang**: Supervision; writing—original draft; writing—review and editing; conceptualization; project administration; funding acquisition.

## CONFLICT OF INTEREST STATEMENT

The authors declare no conflicts of interest.
